# Implementation of the DP-TRANSFERS project in Catalonia: A translational method to improve diabetes screening and prevention in primary care

**DOI:** 10.1371/journal.pone.0194005

**Published:** 2018-03-15

**Authors:** Bernardo Costa-Pinel, Santiago Mestre-Miravet, Francisco Barrio-Torrell, Joan-Josep Cabré-Vila, Xavier Cos-Claramunt, Sofía Aguilar-Sanz, Claustre Solé-Brichs, Conxa Castell-Abat, Victoria Arija-Val, Jaana Lindström, the Catalan Diabetes Prevention Research Group

**Affiliations:** 1 Jordi Gol Primary Care Research Institute, Catalan Health Institute, Primary Health Care Division, Reus-Barcelona, Catalonia, Spain; 2 Public Health Division. Department of Health, Generalitat de Catalunya. Barcelona, Catalonia, Spain; 3 Faculty of Medicine and Health Sciences, Nutrition and Mental Health Research Group (NUTRISAM), Universitat Rovira i Virgili. Institut d’Investigació Sanitaria Pere Virgili. Reus, Catalonia, Spain; 4 Chronic Disease Prevention Unit, National Institute for Health and Welfare, Helsinki, Finland; Florida International University Herbert Wertheim College of Medicine, UNITED STATES

## Abstract

**Background:**

The DE-PLAN-CAT project (Diabetes in Europe–Prevention using lifestyle, physical activity and nutritional intervention–Catalonia) has shown that an intensive lifestyle intervention is feasible in the primary care setting and substantially reduces the incidence of diabetes among high-risk Mediterranean participants. The DP-TRANSFERS project (Diabetes Prevention–Transferring findings from European research to society) is a large-scale national programme aimed at implementing this intervention in primary care centres whenever feasible.

**Methods:**

A multidisciplinary committee first evaluated the programme in health professionals and then participants without diabetes aged 45–75 years identified as being at risk of developing diabetes: FINDRISC (Finnish Diabetes Risk Score)>11 and/or pre-diabetes diagnosis. Implementation was supported by a 4-channel transfer approach (institutional relationships, facilitator workshops, collaborative groupware, programme website) and built upon a 3-step (screening, intervention, follow-up) real-life strategy. The 2-year lifestyle intervention included a 9-hour basic module (6 sessions) and a subsequent 15-hour continuity module (10 sessions) delivered by trained primary healthcare professionals. A 3-level (centre, professionals and participants) descriptive analysis was conducted using cluster sampling to assess results and barriers identified one year after implementation.

**Results:**

The programme was started in June-2016 and evaluated in July-2017. In all, 103 centres covering all the primary care services for 1.4 million inhabitants (27.9% of all centres in Catalonia) and 506 professionals agreed to develop the programme. At the end of the first year, 83 centres (80.6%) remained active and 305 professionals (60.3%) maintained regular web-based activities. Implementation was not feasible in 20 centres (19.4%), and 5 main barriers were prioritized: lack of healthcare manager commitment; discontinuity of the initial effort; substantial increase in staff workload; shift in professional status and lack of acceptance. Overall, 1819 people were screened and 1458 (80.1%) followed the lifestyle intervention, with 1190 (81.6% or 65.4% of those screened) participating in the basic module and 912 in the continuity module (62.5% or 50.1%, respectively).

**Conclusions:**

A large-scale lifestyle intervention in primary care can be properly implemented within a reasonably short time using existing public healthcare resources. Regrettably, one fifth of the centres and more than one third of the professionals showed substantial resistance to performing these additional activities.

## Background

Diabetes is a metabolic disease associated with a great social and economic impact. The global rise in the risk of diabetes–evidenced by the increasing prevalence and incidence of both the disease and related complications–is well-known worldwide. Preventing or delaying diabetes incidence has become a real challenge in all public health systems [1].

The efficacy of clinical trials on lifestyle interventions for diabetes prevention has been well-established [2, 3], and efforts to translate these interventions to primary care settings have shown promising results [4–6]. Long-term projections have also shown that diabetes can be postponed by an average of 5 years in people who already have pre-diabetes [7]. Even if the development of diabetes cannot be prevented but rather only delayed to later in life, this could have a great impact at both an individual and societal level. However, less is known about the real-world effectiveness of large-scale translational efforts since there are several challenges involved in the implementation and evaluation of such tailored lifestyle interventions [4, 8].

The assessment of translation programmes should address three key points: impact on clinical and behaviour indicators commonly associated with a decrease in diabetes risk; programme sustainability by assessing processes carried out and economic analysis of direct costs incurred by the implementation of the programme [9]. Nonetheless, organizational complexity should never mask or justify an unsolved ethical dilemma; if interventions that could prevent or delay the onset of diabetes are available, why should they not reach the target population? An additional reason might be that translation channels do not work properly. In all, this could result in an increase of the consequences of diabetes, a waste of public resources and a loss of potential health benefits [5, 8].

One key example of effectiveness was the European DE-PLAN (Diabetes in Europe–Prevention using lifestyle, physical activity and nutritional intervention) project applied in Catalonia (DE-PLAN-CAT). Diabetes incidence was reduced by 36.5% at the 4-year follow-up in 333 participants carrying out the intensive lifestyle intervention compared to the standard care regime [10]. High-risk participants were first identified with the simple FINDRISC (Finnish Diabetes Risk Score) tool and the core intervention implemented was similar to that applied in the Finnish Diabetes Prevention Study [2, 11] but delivered exclusively by trained primary healthcare professionals following the practice and evidence-based guideline of the IMAGE (Development and Implementation of A European Guideline and Training Standards for Diabetes Prevention) project [12, 13]. When considering economics, a convenient cost-effectiveness ratio (3,243 € per QALY gained) was clearly shown [14]. The project was conducted in 18 primary care centres with an appropriate positive response rate greater than 81% for both screening and intervention. As described in detail previously [15], this model may be feasible and cost-effective in the short-scale in primary care, but if the number of primary care centres and participants is low, a high effect on the community as a whole would not be expected.

The subsequent DP-TRANSFERS (Diabetes Prevention–Transferring findings from European research to society) project was defined and structured as a translational programme aimed at transferring the DE-PLAN-CAT knowledge, methodology, didactic materials and results–if feasible–to daily clinical practice in primary health care. In the near future, this programme will allow assessment of both the widespread effect of this adapted lifestyle intervention and the translational process itself [15]. To our knowledge, this is one of the few existing programmes that has been designed, developed and evaluated entirely in European primary care. Therefore, the present study describes both the preliminary results and barriers encountered along the first year of the implementation process.

## Methods

### Design

The design, objectives, methods, and key indicators used to assess both the results and the process of the entire programme have been published previously [15]. The DP-TRANSFERS project does not have a conventional design since it is a sequential and coordinated set of actions to be performed in primary care in order to achieve a reasonably effective translation of the DE-PLAN-CAT lifestyle intervention using the available resources efficiently.

### Objectives

As for the aims of the programme, the operational objectives are: (1) to establish and expand a multidisciplinary Steering Committee (SC) with representatives from primary care centres to implement a single common translational programme as well as a curriculum for the training of prevention managers (nurses and general practitioners); (2) to identify needs and adapt the lifestyle intervention to the structural conditions in primary care settings which are determinants of real-life clinical practice interventions associated with and predictive of beneficial outcomes; (3) to develop a specific set of easily accessible didactic material in conventional and digital format, and (4) to assess the medium and long-term sustainability and quality of the translation process through the evaluation of resources (balance and cost), actions (intervention effect) and opinion of the target population (facilitators and participants). The development of objectives 1 to 3 started in January-2015. On-site implementation started in June-2016 and a preliminary evaluation was carried out in July-2017 as part of objective 4.

The aims of the present study were to report the findings at one year after the start of implementation, using participation data from the centres (centre level), participation and questionnaire data from service professionals (professional level) and service data on the participants (participant level).

### Organisation, centres and professionals

From January-2015 to May-2016 the implementation of the programme was mainly focused on organisational actions: (1) stratified involvement of primary care centres; (2) improvement of didactic materials; (3) testing of the lifestyle intervention through two pilot groups, and (4) development of the website for exchange of information and data collection. Members of the SC also conducted 30 kick-off briefings (1 hour) for healthcare, resource managers and relevant professionals.

**Fig 1:**
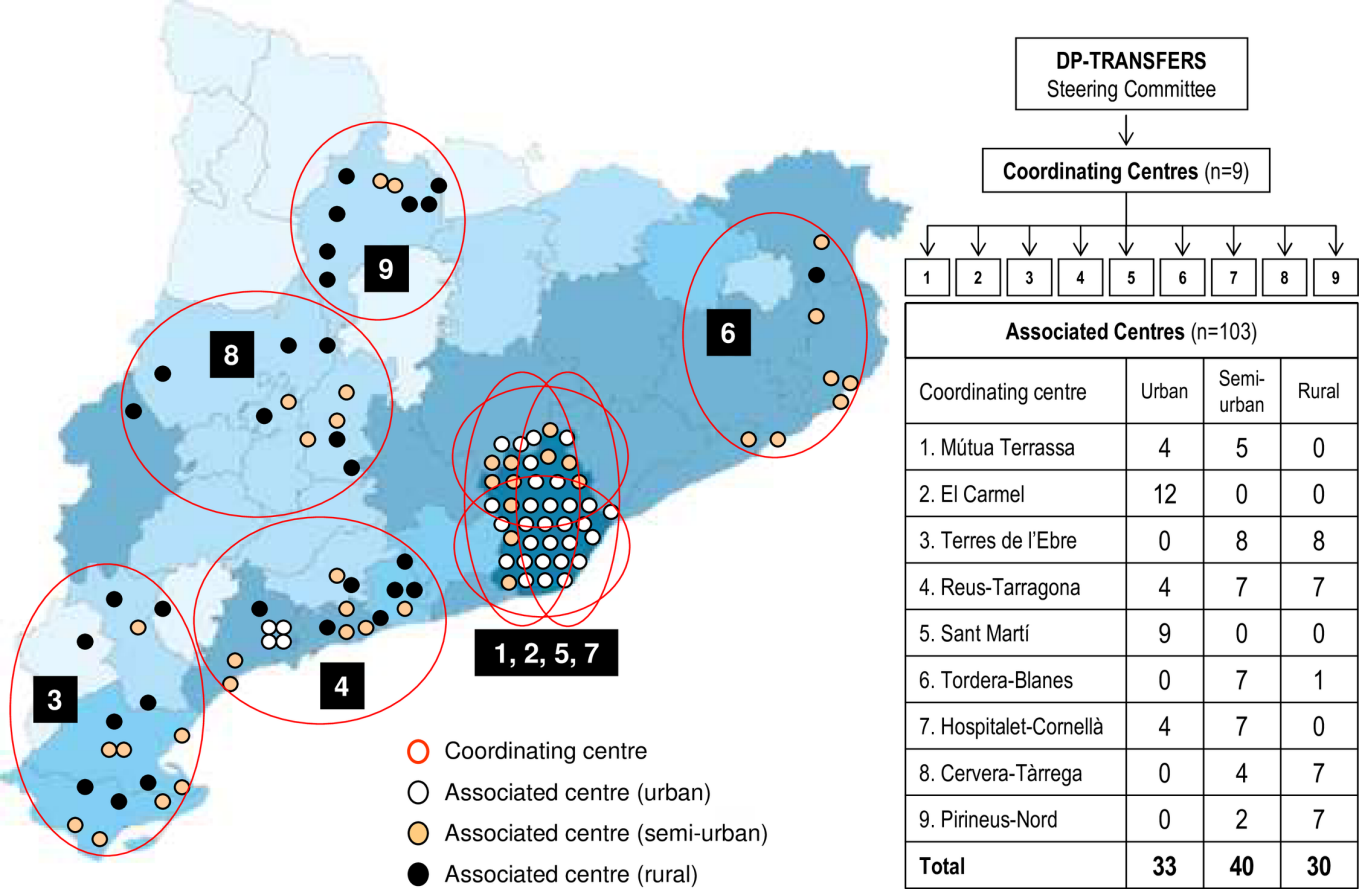
Geographical distribution of the coordinating and associated DP-TRANSFERS centres that participated during the first year of implementation. The colour intensity is proportional to the absolute density of population per county within the Catalan territory.

The project was first addressed to health professionals and then to participants identified as being at risk for diabetes. Involvement of centres and professionals was first agreed upon with the managers and staff on a voluntary basis. The main pre-requisite was the practice having a computerised patient record system. Associated centres were invited in a stratified manner through 9 reference settings (coordinating centres) following a representative national distribution which also took into account particular responsibilities of the centres within the health system (Fig 1). Implementation was supported by a 4-channel transfer approach (institutional relationships, facilitator workshops, collaborative groupware, programme website) and built upon a 3-step (screening, intervention, follow-up) primary care real-life strategy. The main purpose was to integrate the project within the health services portfolio of the maximum number of primary care centres whenever feasible to achieve a non-stop open-loop strategy for diabetes prevention available at all times.

Participating professionals were arbitrarily classified as: (1) coordinators (at least two professionals from each coordinating centre); (2) facilitators (professionals who directly facilitated the lifestyle intervention) and (3) supporters (professionals who collaborated but did not personally facilitate the intervention). A tree structure was developed based on organisation charts proposed by the coordinators who also monitored and periodically reported activities carried out in their associated centres. Particular attention was paid to the development of face-to-face training workshops for professionals in each coordinating centre. Members of the SC provided extensive information about the project including delivery rules, training tools and evaluation criteria during a 5-hour meeting using active participatory methodology.

To assist and define a long-lasting network of professionals, a website was developed (https://www.dp-transfers.cat) which also contained an electronic case report form (eCRF). The main features were: centralised data, easy data logging, fast access to project documents and didactic material, fast internal messaging system and updated consultation at any time. Health professionals proposed by the coordinators received an access password and gave personal commitment by accepting a specific message before logging. Changes in the screening and intervention log, activity within the website and downloading of teaching materials were monitored quarterly from the data centre.

### Target population

Among all users of public primary health care services, the target population corresponds to people without a diagnosis of type 2 diabetes between the ages of 45 and 75 years, with either or both of the following two criteria: (1) Diabetes risk suggested by a FINDRISC score > 11 points [2, 11]; (2) Pre-diabetes diagnosis as defined by the World Health Organization diagnostic criteria [16] based on previous (last year) or current (screening time) laboratory reports: (a) Impaired Fasting Glucose (IFG): fasting plasma glucose (FPG) ≥ 6.1 mmol/l and < 7.0 mmol/l; (b) Impaired Glucose Tolerance (IGT): FPG < 7.0 mmol/l and 2h-postload glucose (2hPG) in the 2-hour 75-g Oral Glucose Tolerance Test (OGTT) ≥ 7.8 mmol/l and < 11.1 mmol/l; (c) IFG plus IGT (both diagnostic categories simultaneously).

All individuals with severe psychiatric disease or serious disorders that could influence the screening or induce discontinuation were excluded at the discretion of the facilitators. Potential participants were contacted by letter, telephone, and text message or otherwise for the first evaluation. If the centre already had another ongoing strategy, computerised clinical records were reviewed in search of eligible participants.

### Lifestyle intervention

**Fig 2:**
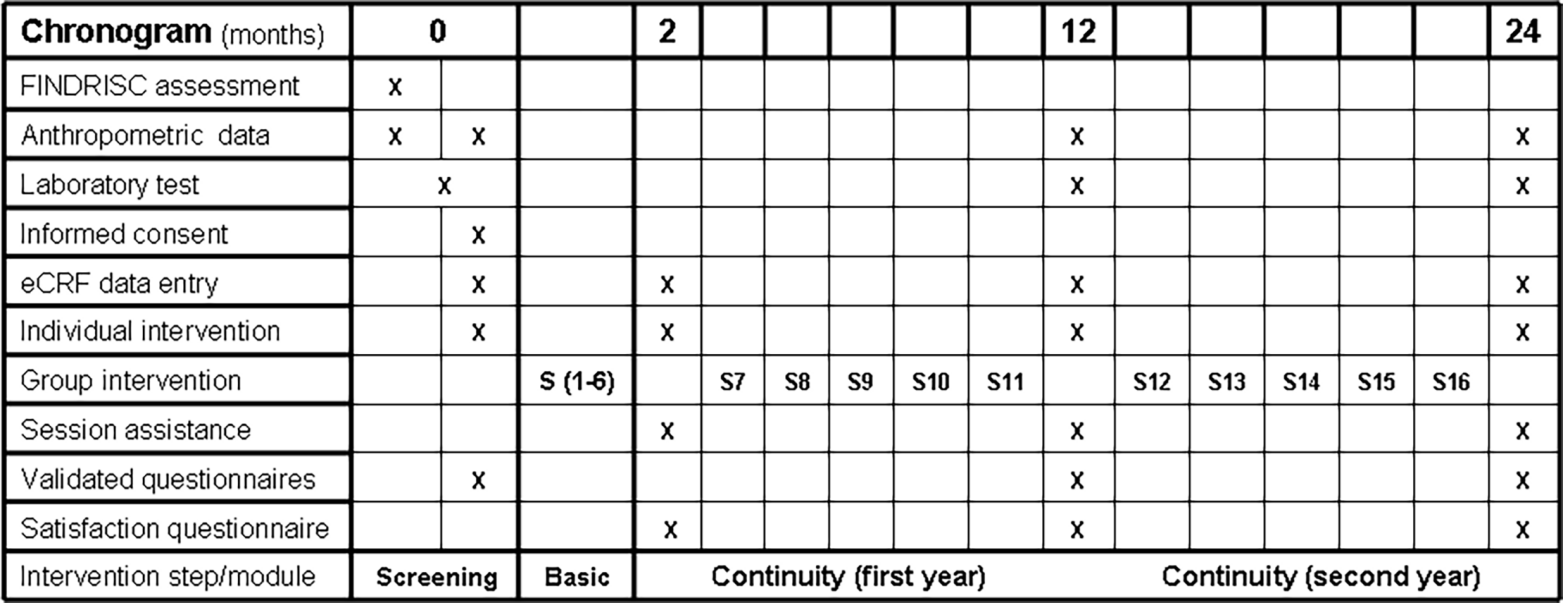
Technical development of the DP-TRANSFERS implementation programme. Abbreviations. FINDRISC: Finnish Diabetes Risc Score; eCRF: electronic Case Report Form; S(n): Group session (number).

The lifestyle intervention was designed by the SC–particularly nursing–and built on two levels: (1) an individual level for personal goal setting, maintenance of motivational support and ways to solve relapses, and (2) a group intervention level to consolidate the changes in habits and behaviours in an attempt to delay disease progression. The core of the 2-year lifestyle intervention consisted of: (1) a 9-hour basic module (6 sessions) delivered during 2 months in groups of 5 to 15 participants receiving specific training materials, and (2) a subsequent 15-hour continuity module (10 sessions) delivered during 22 months as reinforcement of the basic module (Fig 2). The basic module was similar to the intervention provided during the previous reference project (DE-PLAN-CAT) albeit with improvements in the interaction between participants and professionals. The continuity module improved the consistency and extension of the follow-up phase provided by the DE-PLAN-CAT [10].

The contents of the group sessions were clearly addressed through positive messages using active participatory methodology. The intervention materials (identical for each centre) were provided centrally from their own records as well as files adapted from associated European projects on diabetes prevention. The axis of every session consisted of a slide set, activity sheets with homework tasks for participants in addition to specific material for facilitators. Every group session covered relevant aspects of diabetes, cardiovascular risk and are especially aimed at encouraging a healthy relationship towards eating and exercising. The dietary intervention focused on the Mediterranean diet pattern [17]. The physical activity intervention was based on information synthesis and training when feasible.

### Measurements

The measurements and key indicators have been described previously [15]. A set of descriptive indicators referring to the first year of implementation (including the screening step and allocation of the participants in the lifestyle intervention) were selected in view of reporting on the set-up of the programme, referrals and start rates. Members of the SC performed continuous eCRF monitoring and issued a written balance of participation that was sent monthly to all the associated centres. All the participant centres as well as their particular characteristics were clearly identified and recorded in the database.

Professional agreement with the project and didactic materials were evaluated using a 7-item questionnaire specifically designed for the programme (Likert-type scale) that was completed after all face-to-face training workshops by specifying respondent level of disagreement or agreement ranging from 1 (disagreement) to 5 (agreement) points. Additionally, barriers identified after one year of development were prioritised by semi-structured interviews (one-on-one interviews and focus groups) conducted by SC members to obtain feedback from professionals, particularly in centres in which the programme was not feasible. As part of the economic analysis that will be developed at the end of the project, professionals recorded data on specific resources used and extra-time invested in specific activities.

The potentially eligible participants were invited to attend a first examination visit. Information was collected using a structured eCRF, interviewer-administered registration system. As for clinical and anthropometric data, weight, height–body mass index (BMI), waist circumference and blood pressure were measured using standard methods as a part of routine clinical practice. Diabetes risk was assessed by the Catalan/Spanish versions of the FINDRISC, a well-validated 8-item European questionnaire related to diabetes risk factors (ranging from 0 to 26 points) characterising individuals according to their future risk of diabetes as follows: <7 points [low], 7–11 [slightly elevated], 12–14 [moderate], 15–20 [high], >20 [very high] [12]. Participant self-reported interest in introducing lifestyle changes was assessed prior to any intervention. Quality of life was evaluated using a 5-item preference-based health-related quality of life instrument EQ-5D-5L, which is a standardised tool that provides a single index value (ranging from 0 to 100) for health status [18].

Glucose-related parameters (FPG, 2h-PG, HbA1c) and the lipid profile were measured depending on the possibility of determining these values in each centre. It was emphasised that measurements should not increase the burden of laboratory tests but rather should be part of routine practice in primary care. Thus, results available for the last year were also accepted. The plasma glucose and lipid profile determinations were carried out using a uniform glucose oxidase–peroxidase and a cholesterol oxidase–phenol aminophenazone (CHOD-PAP) method, respectively. The HbA1c assay was a standardised HPLC assay aligned to the NGSP (National Glycohemoglobin Standardization Program)–a percentage way of reporting HbA1c values–in all laboratories [19]. The new IFCC (International Federation of Clinical Chemistry) values (mmol/mol) were also calculated. The intra- and interassay coefficients of variation for all assays ranged from 2% to 3%.

### Data collection and analysis

A cluster sampling design was used to select the participant centres (with a probability proportional to the Catalan population size attending primary care), forming a representative sample. The maximum level of participation was determined by 369 pre-existing primary care centres– 334 (90.5%) ascribed to the Catalan Health Institute that served 4.2 million people in 2014 and 35 (9.5%) ascribed to other public providers of health services [20]. The SC approved implementing the project in at least 25% of these centres in order to achieve a significant impact on the whole community.

An effort was made to collect all the data of all the participants in each associated centre through a single centralised eCRF. In centres in which complete data collection was not feasible a minimum consecutive sample of 5, 10 or 15 participants was requested from rural (<5,000 inhabitants), semi-urban (5,000–100,000) or urban (>100,000) strata, respectively. Thus, assuming the worst scenario (all centres are only able to record data on the minimum sample requested) and allowing for a two-year discontinuation rate of close to 20%, it was initially expected to collect data from at least 920 participants (statistical power: 82,5%; type 1 and type 2 errors: 5% and 20%, respectively).

A 3-level (centre, professional and participant) descriptive analysis was performed to assess the preliminary results of participation and the barriers identified one year after programme implementation. Statistical analyses were conducted using SPSS version 15.0 for Windows (SPSS Inc., Chicago, IL, USA). Multiple comparisons of significant differences among groups were carried out by one-way ANOVA and/or by the Student’s t-test for continuous variables and the chi-squared test for categorical variables. The Bonferroni correction was applied when multiple pair wise tests were performed in a single data set. The level of statistical significance was set as p<0.05 for all analyses.

### Ethics approval and consent to participate

The research ethics committee board at the Jordi Gol Research Institute (Barcelona) approved the protocol (January 2015, reference number: P14/141) and each participant signed a written informed consent.

## Results

Table 1 shows an overall summary of involvement by primary care centres, professionals and participants after one year.

**Table 1:** Distribution of participation by coordinating centre at three levels (centres, professionals and participants) one year after implementation.

Analysis level	Primary Care Centres			Primary Care Professionals			Participants		
Coordinating Centre	Proposed	Inactive*	Active	Proposed	Inactive**	Active	Screened	Excluded	Included
**1. Mútua Terrassa**	9	0 (0)	9 (100)	67	22 (32.8)	45 (67.2)	349	54 (15.5)	295 (84.5)
**2. El Carmel**	12	0 (0)	12 (100)	42	9 (21.4)	33 (78.6)	357	90 (25.2)	267 (74.8)
**3. Terres de l’Ebre**	16	2 (12.5)	14 (87.5)	83	37 (44.6)	46 (55.4)	276	44 (15.9)	232 (84.1)
**4. Reus-Tarragona**	18	3 (16.6)	15 (83.4)	64	33 (51.6)	31 (49.4)	240	40 (16.7)	200 (83.3)
**5. Sant Martí**	9	2 (22.2)	7 (77.8)	53	24 (45.3)	29 (54.7)	181	29 (16.1)	152 (83.9)
**6. Tordera-Blanes**	8	1 (12.5)	7 (87.5)	58	25 (45.1)	33 (54.9)	179	64 (35.7)	115 (64.3)
**7. Hospitalet-Cornellà**	11	4 (36.4)	7 (63.6)	83	39 (46.9)	44 (53.1)	144	33 (22.9)	111 (77.1)
**8. Cervera-Tàrrega**	11	0 (0)	11 (100)	53	12 (22.6)	41 (77.4)	84	5 (5.9)	79 (94.1)
**9. Pirineus-Nord**	9	8 (88.8)	1 (11.2)	3	0 (0)	3 (100)	9	2 (22.2)	7 (77.8)
**Total**	103	20 (19.4)	83 (80.6)	506	201 (39.7)	305 (60.3)	1819	361 (19.8)	1458 (80.2)

Data are n or n (%). The coordinating centres are ordered by current number of participants in lifestyle intervention.

(*) Centres without active professionals one year after implementation.

(**) Professionals who did not maintain regular web-based activities.

### Centre-level

In all, 103 centres and rural clinics (27.9% of those active in Catalonia) covering primary care services for 1.4 million inhabitants (Fig 1) gave informed approval for implementing the programme (33 urban, 40 semi-urban and 30 rural). At the end of the first year, 83 centres (80.6%) remained active (31 urban, 32 semi-urban and 20 rural) and 20 (19.4%) had dropped out (2 urban, 8 semi-urban and 10 rural) because of lack of feasibility.

As for the 83 active centres, 44 (53%) included a number greater than or equal to 15 participants (27.3±10.4, range 15 to 54) to receive the lifestyle intervention (corresponding screening range: 17 to 61 individuals). A total of 15 centres (18.1%) included from 10 to 14 (11.1±1.4) participants (corresponding screening range: 10 to 19 individuals) and finally 24 centres (28.9%) included from 5 to 9 (6.4±1.4) participants (screening range: 5 to 20 individuals). The mean centre participation values showed a progressive increase from rural areas (9.1±5.5 participants) to semi-urban (18.8±12.0) and urban areas (25.2±11.8 participants).

None of the 20 centres that discontinued had previously participated in the DE-PLAN-CAT reference project. Of these, 11 centres (55%) maintained sub-optimal screening activities not reaching the minimum sample of participants requested, and 9 centres (45%) failed to screen even the first participant. They were located mainly in dispersed rural areas (n = 10), semi-urban districts with a low-income population (n = 8) and a small proportion corresponded to metropolitan centres (n = 2).

Five main barriers to the development of implementation were discussed and prioritized by the coordinators of the discontinuing centres: (1) lack of commitment of healthcare and resource managers, (2) discontinuity of the initial effort and lack of continuity of commitment by professionals, (3) unsustainable increase in professional workload, (4) shift in professional status, and (5) lack of acceptance of participants or failure to fulfill the inclusion criteria.

### Professional-level

During the first year a password for accessing the website was sequentially sent to 506 professionals: 20 (4%) members of the multidisciplinary Steering Committee (endocrinologist, epidemiologist, dietitian, health technicians and resource managers) and 486 (96%) primary care professionals who agreed to develop the programme at the request of their coordinators, 290 (57.3%) nurses and 196 (38.7%) general practitioners. According to the previously described roles, these professionals were classified as follows: 26 coordinators (5.1%), 361 facilitators (71.3%) and 119 supporters (23.6%).

Nine coordinated kick-off training meetings for professionals–one for each coordinating centre–were organized prior to starting any local intervention (n = 214, mean = 23.7 attendees at each meeting). Facilitator opinion (n = 177) was clearly positive with a mean score of 4.3 out of 5 based on a satisfaction scale ranging from 1 to 5 provided by a 7-item self-administered questionnaire that included technical and motivational items.

The details and the mean score for each question were: (1) Has this experience been positive for you? (4.38); (2) Was the knowledge provided clear and understandable? (4.23); (3) Do you think that the design of this training activity is adequate? (4.26); (4) Do you think the material (slides, presentations) used was understandable and suitable for teaching? (4.37); (5) Was participation in this training activity on preventive intervention profitable? (4.30); (6) Do you think that there was a good relationship among group participants? (4.42) and (7) Would you repeat this experience again? (4.26).

One year after implementing the programme, continuous web-based monitoring showed that 305 (60.3%) professionals (25 coordinators, 226 facilitators and 54 supporters) maintained regular web-based activities (accessing, searching for information, and downloading teaching materials or data entry). Conversely, 201 professionals (39.7%) did not sustain regular activities (1 coordinator, 135 facilitators and 65 supporters), 89 (44.3%) never accessed the website and 112 (55.7%) simply signed up or accessed the website less than once a quarter.

### Participant-level

**Fig 3:**
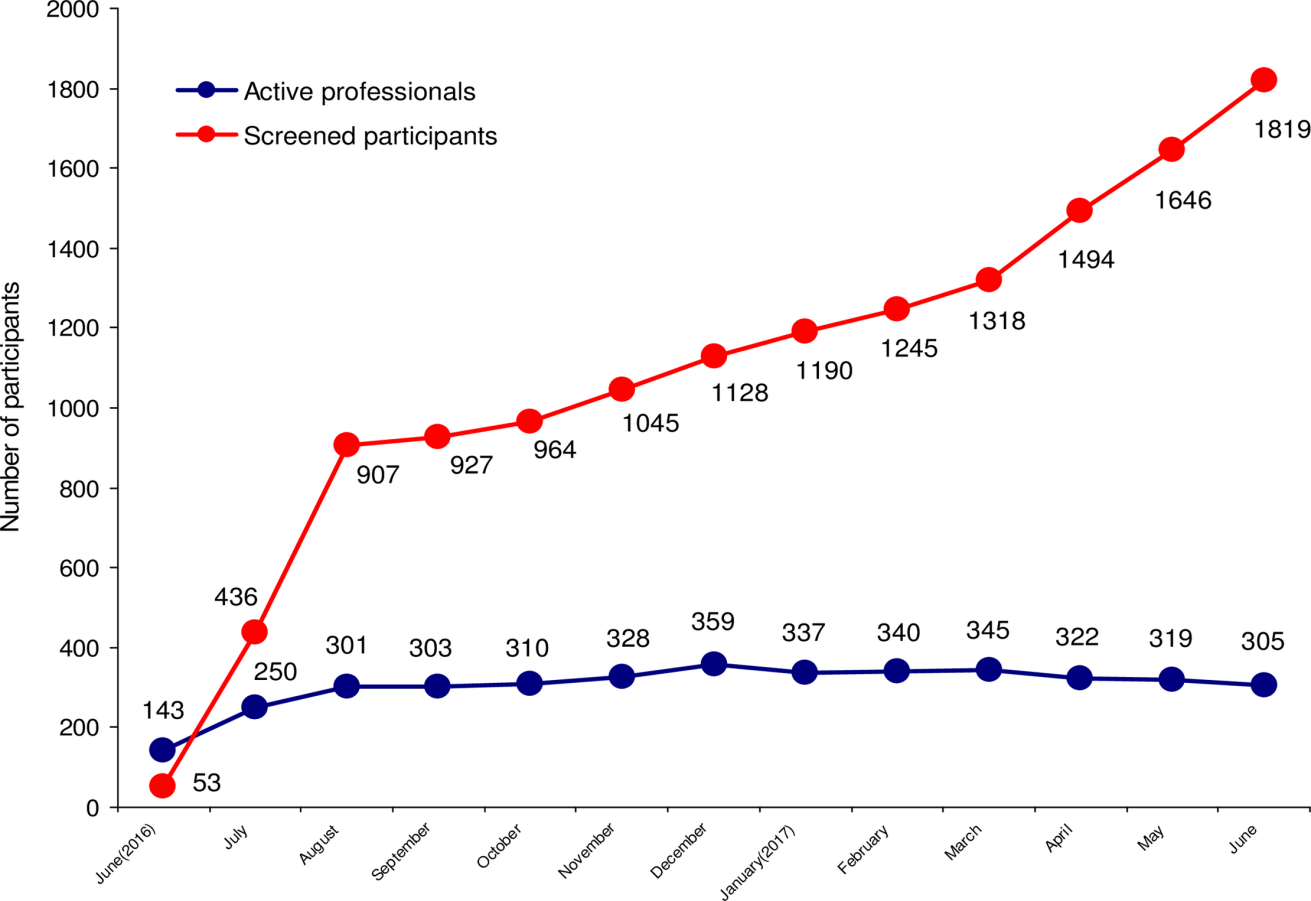
Distribution and monthly progress of programme implementation by active professionals and the participants screened along the first year.

During the first year 1819 individuals were screened, 1458 of whom (80.2%) were accepted to receive the lifestyle intervention and 361 (19.8%) were excluded either for non-compliance with the inclusion criteria, either for personal or technical reasons as detailed below. Table 1 shows the distribution of participation by coordinating centre. Fig 3 depicts the monthly progress by number of participants and professionals. The participation started immediately, reached a maximum slope at the second month, and then continued with a rather modest slope until the end of the first year.

As for the subjects screened, 56.8% were female; the mean age was 63.2 years, and the mean BMI was 31.3 kg/m2. The BMI was higher in women than men (31.8 vs. 30.5 kg/m2, p<0.001) but waist circumference was higher in men (107.4 vs. 102.9 cm, p<0.001). Table 2 shows the characteristics of the baseline risk pattern of participants in the screening step by sex. Diabetes risk was assessed in all cases using the FINDRISC score, and the FPG test while measuring 2hPG was only feasible in 37 subjects (2%). The risk of diabetes assessed by the FINDRISC was higher in women than men (17.4 vs. 17.0 points, p<0.04). In contrast, the risk of diabetes found by the FPG test was higher in men than women (6.2 vs. 6.0 mmol/l, p<0.001). The HbA1c results (n = 898) did not show statistical differences (5.9 vs. 5.8%, p = 0.33). BMI and cholesterol levels were slightly higher in women and systolic and diastolic blood pressure values were discretely higher in men.

**Table 2:** Baseline risk pattern characteristics of participants in the screening step (n = 1819) by sex including FINDRISC and laboratory findings.

Parameter	Overall	Male	Female	*p*
Number of participants (n)	1819	785	1034	
**Age (years)**	63.2±8.0	63.7±7.8	62.8±8.2	0.015
—45-54 y	305(16.8)	114(14.8)	189(18.3)	
—55-64 y	615(33.8)	263(33.5)	352(34.0)	0.092
—≥65 y	899(49.4)	406(51.7)	493(47.7)	
Height (cm)	162.6±9.3	169.6±7.2	157.1±6.7	<0.001
Weight (Kg)	83.1±16.7	88.5±16.4	78.9±15.6	<0.001
**BMI (kg/m**^**2**^**)**	31.3±5.2	30.5±4.5	31.8±5.6	<0.001
—< 25	148(8.1)	58(7.4)	90(9.2)	
—25-30	640(36.8)	324(42.9)	316(32.2)	<0.001
—≥ 30	950(54.7)	374(49.5)	576(60.6)	
**Waist circumference (cm)**	104.9±13.5	107.4±12.8	102.9±13.7	<0.001
—M<94, F<80	95(5.5)	75(9.9)	20(2.0)	
—M 94–102, F 80–88	306(17.7)	209(27.7)	97(9.9)	<0.001
—M ≥103, F ≥89	1330(76.8)	470(62.3)	860(88.0)	
Systolic BP (mmHg)	132.4±13.7	133.8±12.3	131.2±14.4	<0.001
Diastolic BP (mmHg)	78.1±9.2	78.7±9.1	77.5±9.2	0.007
**FINDRISC findings (points)**	17.2±3.6	17.0±3.4	17.4±3.7	0.037
—Low risk (<7)	5(0.3)	1(0.1)	4(0.4)	
—Slightly elevated risk (7–11)	74(4.2)	39(5.2)	35(3.5)	
—Moderate risk (12–14)	330(18.9)	135(17.9)	195(19.8)	0.003
—High risk (15–20)	981(56.3)	455(60.2)	526(53.3)	
—Very high risk (>20)	352(19.4)	126(16.7)	226(22.9)	
**Laboratory findings**				
Fasting Plasma Glucose (mmol/l)	6.1±0.6	6.2±0.6	6.0±0.7	<0.001
2-h Plasma Glucose (mmol/l)	7.7±2.6	7.3±2.7	7.9±2.6	0.493
HbA1c –NGSP (%)	5.9±0.4	5.9±0.4	5.8±0.3	0.336
HbA1c –IFCC (mmol/mol)	41.0±4.4	41.1±4.6	40.9±4.2	0.336
Total Cholesterol (mmol/l)	5.3±0.9	5.1±0.9	5.5±0.9	<0.001
HDL-Cholesterol (mmol/l)	1.5±0.6	1.3±0.5	1.6±0.6	<0.001
LDL-Cholesterol (mmol/l)	3.1±0.9	3.0±0.9	3.2±0.9	<0.001
**Eligibility for lifestyle intervention**				
Included (n)	1458(80.2)	619(78.9)	839 (81.1)	0.226
Excluded (n)	361(19.8)	166(21.1)	195(18.9)	

Data are means ± SD (standard deviation) for quantitative variables or n (%) for qualitative variables. Abbreviations: BMI = Body Mass Index, BP = Blood Pressure, M = Male, F = Female, FINDRISC = Finnish Diabetes Risk Score, IFCC = International Federation of Clinical Chemistry and Laboratory Medicine, NGSP = National Glycohemoglobin Standardization Program.

Table 3 shows the demographic characteristics of the participants in the screening step who were accepted to receive the lifestyle intervention (n = 1458) including the risk pattern and self-reported quality-of-life index and interest in making lifestyle changes. Of these, 516 subjects (35.4%) were classified as being at high type 2 diabetes risk by the FINDRISC, 62 with the blood test (4.2%) or 880 with both tests (60.4%). The risk profile was similar to that described for the whole group. The quality of life index was significantly higher in men who, moreover, showed greater agreement with their present weight and physical activity than women.

**Table 3:** Baseline risk pattern and demographic characteristics of participants in the screening step who ultimately underwent the lifestyle intervention (n = 1458).

Parameter	Overall	Male	Female	*p*
Number of participants (n)	1458	619	839	
Age (years)	63.3±7.8	64.0±7.4	62.7±8.0	<0.001
Height (cm)	162.5±9.4	169.8±7.3	157.0±6.7	<0.001
Weight (Kg)	83.0±16.3	88.3±15.6	79.1±15.7	<0.001
BMI (kg/m^2^)	31.3±5.2	30.4±4.3	32.0±5.6	<0.001
Waist circumference (cm)	105.0±13.3	107.3±12.2	103.0±13.7	<0.001
Systolic BP (mmHg)	132.6±13.7	134.4±12.5	131.2±14.5	<0.001
Diastolic BP (mmHg)	78.2±9.2	78.9±9.2	77.7±9.2	0.014
FINDRISC (points)	17.3±3.6	17.0±3.5	17.55±3.7	0.004
Fasting Plasma Glucose (mmol/l)	6.08±0.6	6.18±0.5	5.99±0.6	<0.001
2-h Plasma Glucose (mmol/l)	7.08±1.7	6.45±1.4	7.48±1.8	0.138
HbA1c –NGSP (%)	5.9±0.4	5.9±0.4	5.9±0.4	0.885
HbA1c –IFCC (mmol/mol)	40.9±4.0	40.9±4.1	40.9±4.1	0.885
Total Cholesterol (mmol/l)	5.3±0.9	5.1±0.9	5.5±0.9	<0.001
HDL-Cholesterol (mmol/l)	1.5±0.6	1.3±0.5	1.6±0.6	<0.001
LDL-Cholesterol (mmol/l)	3.1±0.9	3.0±0.9	3.2±0.9	<0.001
**Diabetes risk (n)**				
—FINDRISC>11	516(35.4)	185(29.9)	331(39.4)	
—Pre-diabetes	62(4.2)	35(5.6)	26(3.1)	<0.001
—Both criteria	880(60.4)	399(64.5)	482(57.5)	
Residence in Catalonia (n≥5y.)	1415(97.0)	599(96.7)	816(97.2)	0.795
Civil status (n, married/couple)	1125(77.2)	514(83.0)	611(72.8)	<0.001
Education (n, basic/secondary)	1262(86.5)	522(84.3)	740(88,2)	<0.001
Smoking (n, never smoker)	742(50.9)	199(32.1)	543(64.7)	<0.001
QoL index*	69.8±19.2	71.7±19.3	68.5±19.0	0.003
Self-reported interest**				
I am happy with my current level of physical activity	2.9±1.5	2.7±1.4	3.0±1.5	0.004
I think my diet is healthy enough	2.6±1.3	2.5±1.2	2.6±1.3	0.551
I am happy with my current weight	3.5±1.4	3.3±1.4	3.7±1.4	<0.001
I am sure that I can make lifestyle changes	2.2±1.3	2.2±1.2	2.2±1.3	0.957

Data are means ± SD (standard deviation) for quantitative variables or n (%) for qualitative variables. Abbreviations: BMI = Body Mass Index, BP = Blood Pressure, FINDRISC = Finnish Diabetes Risk Score, IFCC = International Federation of Clinical Chemistry and Laboratory Medicine; NGSP = National Glycohemoglobin Standardization Program, QoL = Quality of Life.

(*) Single index value provided by the EQ-5D-5L health-related quality of life instrument (ranged from 0 to 100)

(**) Likert-type scale ranged from 1 (agreement) to 5 (disagreement).

As for individuals who were excluded (n = 361), 83 (23%) were ruled out for non-compliance with the inclusion criteria (74 having either FPG, 2hPG or both in the corresponding diabetes interval) and 278 individuals (77%) refused the intervention: 199 (55.2%) withdrew for personal reasons (most alluding to problems in their work planning), 24 (6.6%) signed the informed consent but did not appear in the scheduled intervention group, 26 (7.2%) due to severe personal or family illness, and 29 subjects (8%) with positive screening were excluded because the lifestyle intervention was ultimately not feasible, and they were redirected to another participating centre. Table 4 compares the baseline characteristics of both groups as well as an estimate of the direct resources specifically applied in the screening step. In spite of expected differences in the degree of hyperglycaemia and time spent in screening activities (significantly higher among excluded subjects) no statistical differences were found in the remaining parameters or resources applied between individuals who were excluded or accepted to receive the lifestyle intervention.

**Table 4:** Baseline risk pattern characteristics of participants who were included (n = 1458) or excluded (n = 361) from developing the lifestyle intervention including an estimate of direct resources specifically applied in the screening step during the first year of implementation.

Parameter	Overall	Included	Excluded	*p*
Number of participants (n)	1819	1458	361	
Age (years)	63.2±8.0	63.3±7.8	62.7±9.2	0.55
Sex (% men)	785(43.2)	619(42.5)	166(46.0)	0.22
Height (cm)	162.6±9.3	162.5±9.4	162.9±9.0	0.47
Weight (Kg)	83.1±16.7	83.0±16.3	83.5±18.3	0.67
BMI (kg/m^2^)	31.3±5.2	31.3±5.1	31.0±5.4	0.37
Waist circumference (cm)	104.9±13.5	104.9±13.3	104.7±14.7	0.79
Systolic BP (mmHg)	132.4±13.7	132.6±13.7	131.5±13.4	0.20
Diastolic BP (mmHg)	78.1±9.2	78.2±9.2	77.7±9.2	0.35
FINDRISC (points)	17.2±3.6	17.3±3.6	17.0±3.8	0.08
Fasting Plasma Glucose (mmol/l)	6.1±0.6	6.0±0.6	6.3±0.8	<0.001
2-h Plasma Glucose (mmol/l)	7.7±2.6	7.0±1.7	9.1±3.7	<0.001
HbA1c –NGSP (%)	5.9±0.4	5.9±0.4	5.9±0.5	0.35
HbA1c –IFCC (mmol/mol)	41.0±4.4	40.9±4.0	41.3±5.7	0.35
Total Cholesterol (mmol/l)	5.3±0.9	5.3±0.9	5.3±1.0	0.68
HDL-Cholesterol (mmol/l)	1.5±0.6	1.5±0.6	1.5±0.6	0.96
LDL-Cholesterol (mmol/l)	3.1±0.9	3.1±0.9	3.0±0.9	0.23
**Estimation of direct resources**				
**Average time spent in scheduled activities** (minutes)				
—History review	9.5±5.2	9.5±5.1	9.6±5.2	0.73
—Obtaining informed consent	6.1±3.7	5.8±3.5	6.3±3.8	0.004
—Filling out the FINDRISC	5.0±2.9	4.9±2.8	5.1±3.0	0.15
—Completing the eCRF	10.6±8.0	10.5±8.2	10.8±8.0	0.39
—Establishing contacts and appointments	5.1±4.3	5.9±4.1	5.3±4.5	0.11
—Scheduling visits	13.7±11.8	13.3±11.9	14.1±11.7	0.17
—Scheduling laboratory test	4.7±8.4	4.6±8.9	4.8±7.9	0.68
—Total	54.1±24.6	52.6±24.0	55.1±25.0	0.03
**Blood test**				
—Already available (n)	1521(83.6)	1204(82.6)	317(87.8)	0.09
—Specifically performed (n)	298(16.4)	254(17.4)	44(12.2)	
Phone calls (n)	1.4±0.9	1.5±1.0	1.4±0.9	0.24
Text messages (n)	0.04±0.3	0.04±0.3	0.03±0.2	0.23
Specific visits (n)	1.4±0.7	1.4±0.7	1.4±0.6	0.61
Participants receiving printed material (n)	258(14.8)	123(16.2)	135(13.7)	0.13
Average of sheets delivered (n)	3.8±3.0	3.8±3.1	3.7±2.9	0.56
**Staff participation (proportion)**				
—Nursing staff (%)	75.6±35.9	75.1±36.5	75.8±35.5	0.64
—Medicine staff (%)	24.0±35.8	24.4±36.3	23.5±35.5	0.68
—Administrative staff (%)	0.6±3.4	0.5±2.5	0.7±3.9	0.16

Data are means ± SD (standard deviation) for quantitative variables or n (%) for qualitative variables. Abbreviations: BMI = Body Mass Index, BP = Blood Pressure, M = Male, F = Female, FINDRISC = Finnish Diabetes Risk Score, IFCC = International Federation of Clinical Chemistry and Laboratory Medicine. NGSP = National Glycohemoglobin Standardization Program, eCRF = Electronic Case Report Form.

During the first year of implementation 130 groups of participants received the basic module of the lifestyle intervention (sessions 1 to 6), 100 of which had completed the module and 30 were pending completion. A total of 93 groups reached the continuity module (sessions 7 to 11): 18 of which were completed and 75 ongoing. Overall, 1190 (81.6%) individuals who were accepted to receive the lifestyle intervention (65.4% of those screened) had already participated in the basic module and 912 had done the continuity module (62.5% and 50.1%, respectively). Although an in-depth analysis is currently not available, the number of participants in the basic module tended to be higher than in the continuity module.

## Discussion

The feasibility and effectiveness of lifestyle interventions need to be validated within the population in which they are intended to be used. The Catalan DE-PLAN project clearly showed that the overall incidence of diabetes could be reduced in high-risk individuals following the intensive intervention compared with the standard care regime [10]. This was a significant result with important implications for primary healthcare-based diabetes prevention. However, the real challenge was to translate this intervention into the daily clinical practice of the largest number of primary care settings possible whenever feasible.

The implementation of a translational diabetes prevention project in primary care settings is a complex and multi-level process involving centres, professionals as well as individuals who contact public health services. The DP-TRANSFERS project combines research, training and teaching activities with primary healthcare practice. If implementation refers to the adequacy of results to primary care, this project is working properly with a favourable opinion of professionals and large-scale participation.

One of the strengths of this project was the continued management by a multidisciplinary committee which even included monitoring the activity within the project website. Based on this information, coordinators could periodically assess whether their staff remained active or not, provide positive feedback and suggest changes in primary care teams or centres, as needed. In this way, shifts in professional status, sick leave and other reasons for sustained inactivity could be recognized and solved when feasible. Obviously, the findings on access to the website by health professionals are not the only information needed to determine the extent to which professionals were engaged with the programme or about service delivery. Moreover, not all professional activity is reflected in the electronic data collection form or on the website, which is a limitation. Accordingly, we cannot rule out some bias, and it is likely that the real participation may have been underestimated.

Nevertheless, one fifth of the centres and more than one third of the professionals showed substantial resistance to performing these additional activities. Professionals were specifically trained in the methodology needed to achieve the objectives but they are still far from the recently proposed academic qualification of translational process experts [21]. Although there is already a critical mass of professionals who keep the project active, there are 3 relevant challenges at work: (1) at one-year of follow-up it is likely that only half of the primary care centres have successfully integrated the scheduled activities into routine clinical practice, (2) a significant number of professionals identified the project as a conventional study, and therefore, did not achieve the required level of participation to keep the programme active over time, (3) a non insignificant number of professionals will probably leave the project if they are not encouraged [22, 23]. As to date the project intended to use only existing public resources, the most appropriate response would probably be to increase the funds allocated [24]. However, it could also be useful to include DP-TRANSFERS activities within the contractual professional objectives whether academic, economic or both. Both possibilities are currently being discussed through regular contacts with resource managers.

This one-year preliminary report precedes a more complex data analysis but reveals the risk profile of the population screened and the trends of participation. Two relevant facts should be highlighted. First, the number of participants screened far exceeded the calculated estimates. Second, the risk profile was similar to that of the general population attending primary care. As shown previously, women are most likely to use these services [25], and this predominance is comparable to previous large trials concerning diabetes prevention [2, 3]. Similar to these trials, the number of men in our project was lower than the number of women, and the proportion of men aged over 65 years was slightly greater than the proportion of women of the same age included. In any case, the population screened was at high risk for not only diabetes but also for cardiovascular disease. Apparently it could be argued that the implementation and sustainability on time worked better in urban and semiurban areas. However, the 10 rural centres that failed (total population less than 2000 inhabitants) were located in geographically dispersed areas not always well communicated–even by road–which is an additional barrier to implementation.

We are aware that this type of intervention could be questionable in terms of accurately reaching the population most in need [8]. Moreover, one fifth of the individuals screened did not perform the intervention and more than half referred to personal problems, particularly in their work schedule. Direct comparison of compliers and non-compliers, without the non-eligible cohort would probably be more informative, but the sample studied was too small to draw conclusions. As a result, it is very clear that the programme should be extended for many years to reach a relevant proportion of the target population in order to share possible benefits by society at large. Although there are costs associated with such a prevention programme, an average of 54 minutes spent per participant and just 16% of new laboratory tests specifically performed to implement the screening (since 84% of them were already available) are promising rates regarding future cost-effectiveness analysis. Meanwhile, assuming that screening is cheaper than lifestyle intervention as a whole; a persuasive internet-based facilitation system is being developed and should soon be available.

Similarly to most well designed clinical and implementation initiatives, traditional lifestyle intervention modes were used such as individual and group counselling. As usual, the efficiency may vary depending on the dietary habits and the ability of an intervention to significantly reduce weight. In contrast, the results of the PREDIMED (Prevention with Mediterranean diet) study have shown that a non–energy-restricted traditional Mediterranean diet (usually high in unsaturated fat) can be a useful tool for preventing diabetes [17]. Actually, more research is needed to clarify the mechanisms leading to a reduction in diabetes risk independently of weight loss but education of the population on the Mediterranean diet combined with regular exercise might also be a safe public health approach to delay or prevent the development of diabetes in Catalonia.

Overall participation increased over time, albeit not proportionally. While the increase in participation continued it slowed down after the third month achieving a stable range of active professionals which was sufficient to carry out the successive steps of the programme. Hypothetically, the first two-month period corresponds to an elastic phase that only depends on the number of centres, professionals and resources invested. The second period represents a plastic phase that could only continue without a rupture as long as remodelling of the participating primary care teams is guaranteed. Achieving a convenient translational balance between these two stages would define the tractability of the programme and its options for real strengthening over time.

## Conclusions

This study presents the results and barriers of a large-scale, primary care-based diabetes prevention initiative in Catalonia and provides some insight into practical successes and challenges in scaling up an evidence-based lifestyle intervention in 103 primary care settings. Quantitative data is also provided, which is an uncommon finding within the existing scientific literature on this subject.

The present results demonstrate that implementing a large-scale lifestyle intervention in primary healthcare is feasible and can be properly launched within a reasonably short time using existing public healthcare resources. This information could be useful for practices, researchers, and policy-makers interested in the implementation of translational programmes on type 2 diabetes prevention.

Regrettably, one fifth of the centres and more than one third of the professionals showed substantial resistance to performing these additional activities. Undoubtedly, the main reason was that the programme was conducted under real working conditions. More in depth studies are needed to increase the scope, and evaluate the methodological issues surrounding the implementation and comparison of the project with others also carried out under standard care conditions.

## Abbreviations

DE-PLAN: Diabetes in Europe–Prevention using lifestyle, physical activity and nutritional intervention
FINDRISC: Finnish diabetes risk score
PREDIMED: Prevention with Mediterranean diet
DP-TRANSFERS: Diabetes Prevention–Transferring findings from European research to society
SC: Steering Committee
eCRF: electronic case report form
IFG: Impaired fasting glucose
FPG: Fasting plasma glucose
IGT: Impaired glucose tolerance
2hPG: 2h-Postload glucose
OGTT: Oral glucose tolerance test
